# Line and V-Shape Formation Based Distributed Processing for Robotic Swarms

**DOI:** 10.3390/s18082543

**Published:** 2018-08-03

**Authors:** Jian Yang, Xin Wang, Peter Bauer

**Affiliations:** 1 Department of Mechanical and Automation Engineering, Harbin Institute of Technology Shenzhen, Shenzhen 518055, China; yangjian_hit@yahoo.com; 2 Department of Electrical Engineering, University of Notre Dame, Notre Dame, IN 46656, USA; pbauer@nd.edu

**Keywords:** swarm robotics, sensor networks, pattern formation, distributed processing, collaborative exploration

## Abstract

Efficient distributed processing is vital for collaborative searching tasks of robotic swarm systems. Typically, those systems are decentralized, and the members have only limited communication and processing capacities. What is illustrated in this paper is a distributed processing paradigm for robotic swarms moving in a line or v-shape formation. The introduced concept is capable of exploits the line and v-shape formations for 2-D filtering and processing algorithms based on a modified multi-dimensional Roesser model. The communication is only between nearest adjacent members with a simple state variable. As an example, we applied a salient region detection algorithm to the proposed framework. The simulation results indicate the designed paradigm can detect salient regions by using a moving line or v-shape formation in a scanning way. The requirement of communication and processing capability in this framework is minimal, making it a good candidate for collaborative exploration of formatted robotic swarms.

## 1. Introduction

Searching large areas for a particular signal or feature is a formidable task that, at the moment, has no satisfactory solution. Typical approaches include the use of very few high complexity robotic devices that randomly explore the area of interest in a sequential fashion [1]. The disadvantages of such approaches are apparent: long search time, high probability of missing the target, high cost, and the possibility of loss of the agent, which again adds to the expected cost value. Also, to keep the detection probability high enough, one has to pursue a large number of false positives, further adding to the time cost for such a scenario. Using multi-robot systems to perform such tasks in parallel will be a better choice.

Swarm robotics is a particular approach for multi-robot systems that takes inspiration from the self-organized behaviors of social animals such as birds, bees and fish groups, etc. [2]. Those systems are generally with a scalable decentralized scheme that the members do not have any global knowledge, and only have local sensing and limited communication capability [3]. This area has been perceived to have relevance in a variety of application areas ranging from exploration of virgin territories (mid-air [4] or underwater [5]), area coverage for military defense to contamination detection or tracking [6], etc. Ordinarily, the members in swarms are homogeneous. However, some complex applications require to use multiple types of sensors and robots simultaneously, all of which could not be integrated into a single type of agent. Those applications, therefore, require the system have some heterogeneity [7]. Depending on the requirements of different tasks, it may need physically heterogeneous members, or the heterogeneity in behavior could be achieved in physically homogeneous teams by specializing the behaviors [8].

In this paper, we are adopting robotic swarms for collaborative searching tasks. To cope with this, the swarms must first move together and then make a decision together under the condition of limited sensing and communication [9]. One conventional approach is searching randomly in an area to get the optimal target location. This idea is also the basis of particle swarm optimization (PSO) algorithm [10], which have many extensions of imitating species including ants, fish groups as well as glowworms [11]. However, robots in those solutions in an open area have the high probability of losing contact with each other and, therefore, lose the ability to finish the task [12]. By forming a specific formation and moving together to search an unknown area in a scanner-like way is a better option for those tasks.

Formation forming is one of the fundamental research topics of swarm collaborative behaviors. It aims at deploying robots regularly and repetitively that keeps specific distances from each other to get the desired pattern during moving. There are numbers of research works dealing with formation control problems in literature: structure-based methods (leader-follower [13], virtual structure [14]), behavior-based approaches (finite state machine [15], potential fields [16], consensus-based methods [17]) as well as multicellular mechanism-inspired paradigms [18]. In particular, flying geese inspired line or v-shape formation control is well-studied in this area. It is easy to deploy and has the significant potential for searching an area in a cooperative and parallel way. Sousselier et al. proposed a line formation algorithm for the underwater environment searching task [1]. Nathan et al. introduced a forming method which employed a small set of distributed positioning rules, i.e., coalescing, gap-seeking and stationing rules, to guide the movements of artificial flocking agents aimed to get the v-like formation [19]. Li et al. demonstrated a v-shape formation method from control engineering standpoint. By combining the visual communication constraints and a cost function, a gradient-based navigation control algorithm was given for the forming task [20]. However, applying those formations to collaborative searching tasks require processing the sampled data instantly during moving. Especially the swarm member is under the condition of local communication and limited processing capacity.

In fact, the event searching scheme with those formations requires fast processing capability. Since the system is designed to be decentralized, uploading the sampled and possibly pre-processed data to an entity outside the swarm and then communicating the decision back to the appropriate swarm agents is simply not an option. Fast, in-swarm distributed processing is essential for timely decision making to increase the ability to avoid false negatives at the cost of allowing false positives. This paper illustrates an in-swarm distributed processing paradigm for flying geese inspired line and v-shape formations that have the capability of covering and exploring vast areas effectively. We have shown a formation-based method for collaborative decision making in a scanner-like way of a low-complexity robots swarm with distributed processing paradigm [21]. In this work, formation formed swarm was treated as a moving sensor network, in which could utilize some multi-dimensional-based algorithms in a distributed manner [22]. We extended the work to more general cases that address not only the line formation case but also the v-shape case on both distributed signal processing and collaborative decision making aspects. An example of distributed salient region detection algorithm is applied to this paradigm to show the effectiveness.

The main contributions of this paper are the real-time realization of a modified 2-D Roesser model for in-swarm distributed processing of line and v-shape formations. This work extends the results in [22] that only work for rectangular static 2-D sensor networks. The approach allows detecting signals of interest during motion without communicating data into or out of the swarm. A salient region detection example is given to show the effectiveness of the proposed paradigm.

## 2. Problem Statements

**Figure 1:**
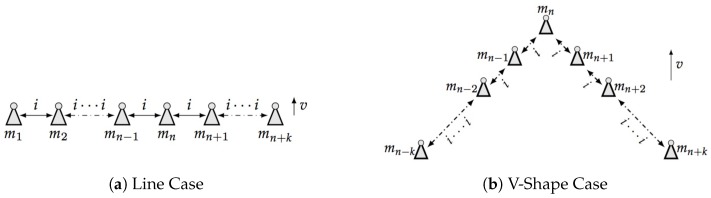
Desired Distributed Processing Procedure.

We are proposing to develop a distributed processing scheme that assigns one swarm agent per sampling point and processes data using simple communication with only nearest neighbors. As shown in Figure 1, suppose the line and v-shape formation are already created and moving perpendicular to the direction of formed patterns (*v* in the Figure). By constraint the information *i* is only exchanged between closest neighbors, denote $u_{n}$ and $y_{n}$ are input and output of moving node *n*, the distributed processing problem can be written as follows:

(1) $$y_{n}\left(t\right)=f\left[u_{n}\left(t\right),i_{n-1}\left(t\right),i_{n+1}\left(t\right),i_{n}\left(t-1\right)\right]$$

where $i_{t}\left(n-1\right)$ and $i_{t}\left(n+1\right)$ are signals received by node *n* from the previous and next adjacent member in the swarm respectively. $i_{t-1}\left(n\right)$ is the stored self-state of last time slot. That means the system has propagation causality in the directions of signal transmission. Here we assume that the signals that need to be exchanged are simple, and can be transmitted by radio or visual beacons without packet loss. Now the distributed processing problem translates to how to apply suitable filters into this scheme depending on task requirements.

## 3. Methods

Due to its many advantages, we propose a modified 2-D Roesser model for distributed processing problem under the line and v-shape formations. This two-dimensional processing model, initially designed for centralized (image) processing, has many features that are of particular interest for the problem at hand: scalability, high network capacity, and low node complexity.

### 3.1. Standard 2-D Roesser Model

There are several causality structures (and therefore directions of propagation) that can be chosen within this framework. The choice of the structure depends on different aspects of swarm operations and is discussed in more details later on. For reasons of simplicity, we initially introduce the case of “quarter plane causal” of a standard 2-D model, which can easily be transformed to other regions of support, and then present the modified model for line and v-shape processing case.

**Figure 2:**
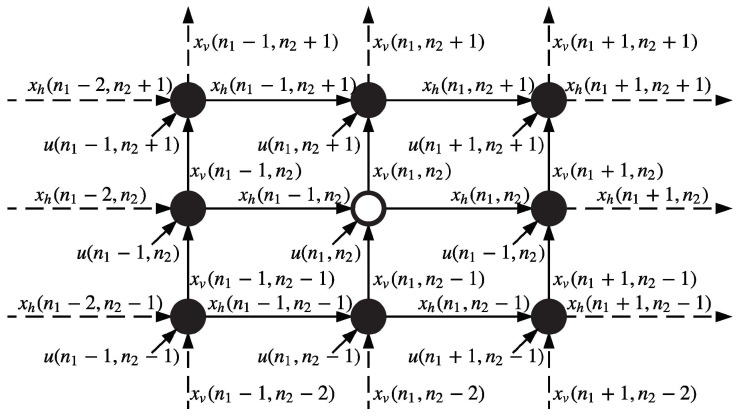
Standard 2-D Roesser model with first quadrant causality.

In its most basic, linear form, the 2-D Roesser state space model is given by [23]:

(2a) $$\begin{array}{cc} \left[\begin{array}{c} x_{h}\left(n_{1}+1,n_{2}\right)\\ x_{v}\left(n_{1},n_{2}+1\right) \end{array}\right] & =\left[\begin{array}{cc} A_{11} & A_{12}\\ A_{21} & A_{22} \end{array}\right]\left[\begin{array}{c} x_{h}\left(n_{1},n_{2}\right)\\ x_{v}\left(n_{1},n_{2}\right) \end{array}\right]+\left[\begin{array}{c} B_{1}\\ B_{2} \end{array}\right]u\left(n_{1},n_{2}\right) \end{array}$$

(2b) $$\begin{array}{cc} y(n_{1},n_{2}) & =\left[\begin{array}{cc} C_{1} & C_{2} \end{array}\right]\left[\begin{array}{c} x_{h}\left(n_{1},n_{2}\right)\\ x_{v}\left(n_{1},n_{2}\right) \end{array}\right]+Du\left(n_{1},n_{2}\right) \end{array}$$

where ${\left(x_{h}\left(n_{1},n_{2}\right),x_{v}\left(n_{1},n_{2}\right)\right)}^{T}$ is the state vector, $x_{h}$ and $x_{v}$ are the horizontal and vertical propagating components, $u(n_{1},n_{2})$ and $y(n_{1},n_{2})$ are the input and output signals of the filtering process, respectively. The direction of response propagation is shown in Figure 2. The center node $\left(n_{1},n_{2}\right)$ in the figure receives the state information from only the closest adjacent nodes horizontally and vertically (see $x_{h}\left(n_{1}-1,n_{2}\right)$ and $x_{v}\left(n_{1},n_{2}-1\right)$ in the figure). By combing with its own sample input $u(n_{1},n_{2})$, the node is able to compute its output $y(n_{1},n_{2})$ as well as the transmission status $x_{h}\left(n_{1},n_{2}\right)$ and $x_{v}\left(n_{1},n_{2}\right)$ respectively. Those status again propagate to next neighbor nodes $\left(n_{1}+1,n_{2}\right)$ and $\left(n_{1},n_{2}+1\right)$.

While the Roesser model in its original form clearly creates first quadrant causality, other options exist. For example on a square lattice, four different quarter-plane causalities correspond to the four different diagonal signal propagation directions, that in turn can be combined to generate the total outputs. The following three causalities exist for a 2-D quarter-plane by changing the left-hand side of Equation (2a) to $\left[\begin{array}{c} x_{h}\left(n_{1}-1,n_{2}\right)\\ x_{v}\left(n_{1},n_{2}+1\right) \end{array}\right]$, $\left[\begin{array}{c} x_{h}\left(n_{1}-1,n_{2}\right)\\ x_{v}\left(n_{1},n_{2}+1\right) \end{array}\right]$ and $\left[\begin{array}{c} x_{h}\left(n_{1}+1,n_{2}\right)\\ x_{v}\left(n_{1},n_{2}-1\right) \end{array}\right]$, which cooresponding to 2nd, 3rd and 4th quadrant causalities respectively.

By comparing the standard Roesser model and the distributed processing problem we modeled in Equation (1), we found that the two models have similarities. If we replace $i_{n-1}\left(t\right)$, $i_{n+1}\left(t\right)$ and $i_{n}\left(t-1\right)$ in (1) with the corresponding horizontal status $x_{h}$ and vertical status $x_{v}$ in (2), the distributed processing problem will be solved by transmitting the status variable with certain propagation causalities defined above. The work in [22] provides a distributed processing model for the case of a fixed rectangular two-dimensional lattice of a sensor network. Since the full 2-D sampling lattice is not available at any given time for moving swarm in formations, one cannot perform the algorithm in (2) as outlined in Figure 2. Therefore modified versions need to be formulated for moving line or v-shape cases where the agents need to remember the state of the previous time instant to simulate the vertical status propagation.

### 3.2. Modified 2-D Roesser Model Based Distributed Processing

**Figure 3:**
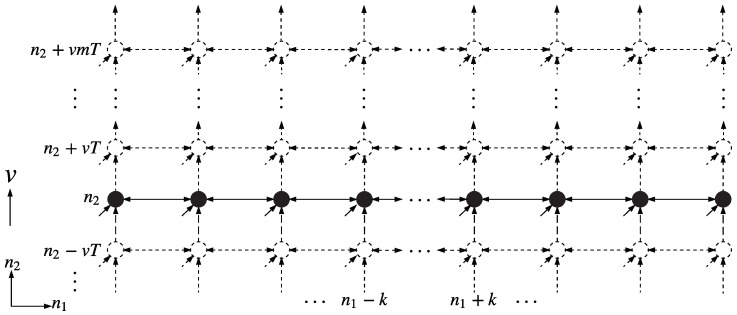
Roesser Model Based Distributed Processing in 1-D Case.

Assume for a moment the line-formatted swarm motion is in the direction of the $n_{2}$ axis in Figure 3. Then all sampling lattice locations $\left(n_{1},n_{2}\right)$, $n_{2}=0,1,2\ldots$ with $n_{1}$ fixed are represented by the same swarm agent, uniquely identified as agent $n_{1}$. Sampling at location $\left(n_{1},n_{2}\right)$ then just happens *t* time units $\left(t=n_{2}/v\right)$ after sampling at $\left(n_{1},0\right)$, where *v* is the agent speed in the $n_{2}$ direction, and agent speed is defined merely as sampling periods per time. In this case, only the horizontal part of Equation (2a) requires communication between neighboring agents. All the computations are executed entirely inside each line agent. Therefore this scheme (unlike the 2-D process before) has a sampling and processing rate limit that is dictated by the swarm speed itself. A slow-moving swarm takes more time to arrive at the next sampling instant $n_{2}$ and thus has plenty of time for propagating the state value from left to right, vice versa, or in both $\pm n_{1}$ directions. The question of causality choice is almost trivial for this case, only in the first and second quadrant, since the swarm does not have any information about the un-scanned area.

By assigning $n_{1}$ as a member among the swarm, the left part members are $\left(n_{1}-1,\ldots ,n_{1}-k\right)$ while the right part members are $\left(n_{1}+1,\ldots ,n_{1}+l\right)$, the processing procedure of v-shape case and line case are similar in most respects. The shape of the coverage area may differ according to the final formation of the swarm. Details will be shown in the following sections of results and discussions.

### 3.3. Distributed Salient Region Detection

Salient region detection can be utilized to perform fast scene analysis and target tracking. Here we assume the formations will be used to detect salient regions of an unknown area. In this example, suppose every single agent can acquire image sequences and calculate some simple features, such as average color weight or intensity, which is treated as the input of each agent. According to the center-surrounding mechanism [24], saliency values for those features should be the differences between the current region and its neighbors. The difference equations of this mechanism for a $N_{1}\times N_{2}$ filter with first quadrant causality can be written as:

(3) $$y\left(n_{1},n_{2}\right)=|\sum_{k_{1}=0}^{N_{1}-1}\sum_{k_{2}=0}^{N_{2}-1}u\left(n_{1},n_{2}\right)-\frac{u(n_{1}-k_{1},n_{2}-k_{2})}{N_{1}\times N_{2}}\left|=\right|\tilde{y}\left(z_{1},z_{2}\right)|$$

where *u* and *y* are the inputs and outputs respectively. Let $N_{1}=N_{2}=2$, the following filter in its *z* transform can be established for the transfer function above:

(4) $$H\left(z_{1},z_{2}\right)=\frac{\tilde{y}\left(z_{1},z_{2}\right)}{u(z_{1},z_{2})}=-\frac{1}{4}z_{1}^{-1}-\frac{1}{4}z_{2}^{-1}-\frac{1}{4}z_{1}^{-1}z_{2}^{-1}+\frac{3}{4}$$

Based on the multidimensional system theory [25], one corresponding Roesser state space model for this transfer function in the first and second quadrant causality is written as (5) and (6) respectively.

(5a) $$\begin{array}{cc} \left[\begin{array}{c} x_{h}\left(n_{1}+1,n_{2}\right)\\ x_{v}\left(n_{1},n_{2}+1\right) \end{array}\right] & =\left[\begin{array}{cc} 0 & 0\\ -\frac{1}{4} & 0 \end{array}\right]\left[\begin{array}{c} x_{h}\left(n_{1},n_{2}\right)\\ x_{v}\left(n_{1},n_{2}\right) \end{array}\right]+\left[\begin{array}{c} 1\\ -\frac{1}{4} \end{array}\right]u\left(n_{1},n_{2}\right) \end{array}$$

(5b) $$\begin{array}{cc} y_{1}\left(n_{1},n_{2}\right) & =|\left[\begin{array}{cc} -\frac{1}{4} & 1 \end{array}\right]\left[\begin{array}{c} x_{h}\left(n_{1},n_{2}\right)\\ x_{v}\left(n_{1},n_{2}\right) \end{array}\right]+\frac{3}{4}u\left(n_{1},n_{2}\right)| \end{array}$$

(6a) $$\begin{array}{cc} \left[\begin{array}{c} x_{h}\left(n_{1}-1,n_{2}\right)\\ x_{v}\left(n_{1},n_{2}+1\right) \end{array}\right] & =\left[\begin{array}{cc} 0 & 0\\ -\frac{1}{4} & 0 \end{array}\right]\left[\begin{array}{c} x_{h}\left(n_{1},n_{2}\right)\\ x_{v}\left(n_{1},n_{2}\right) \end{array}\right]+\left[\begin{array}{c} 1\\ -\frac{1}{4} \end{array}\right]u\left(n_{1},n_{2}\right) \end{array}$$

(6b) $$\begin{array}{cc} y_{2}\left(n_{1},n_{2}\right) & =|\left[\begin{array}{cc} -\frac{1}{4} & 1 \end{array}\right]\left[\begin{array}{c} x_{h}\left(n_{1},n_{2}\right)\\ x_{v}\left(n_{1},n_{2}\right) \end{array}\right]+\frac{3}{4}u\left(n_{1},n_{2}\right)| \end{array}$$

where $x_{h}\left(n_{1},n_{2}\right)$ and $x_{v}\left(n_{1},n_{2}\right)$ are horizontal and vertical state variables respectively. The salient value can be assigned to a scanned region by combining the two directions of propagation causalities:

(7) $$y\left(n_{1},n_{2}\right)=y_{1}\left(n_{1},n_{2}\right)+y_{2}\left(n_{1},n_{2}\right)$$

## 4. Results

**Figure 4:**
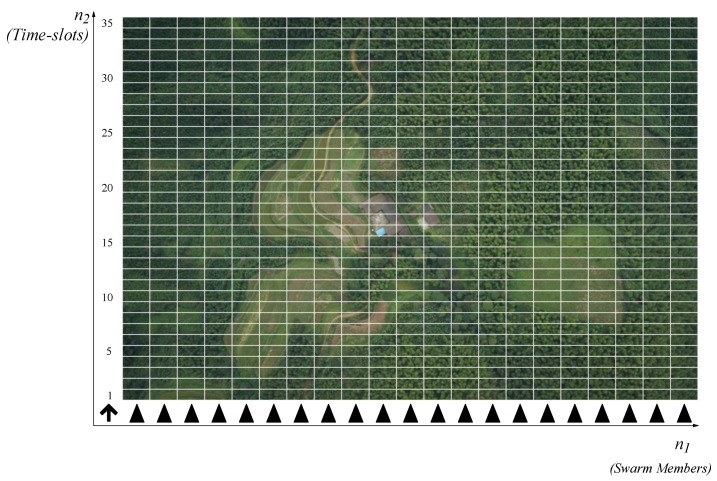
Simulation Configuration of Line Scan Case.

For line case, we split a large scene to 21×35 blocks to simulate distributed sampling and processing procedure. As shown in Figure 4, this segmentation simulates 21 robots formatted a line in $n_{1}$ axis, moving in $n_{2}$ direction and processing in 35 consecutive time slots. The simulation dataset consists of 222 large scenes of forest captured by unmanned aerial vehicle (UAVs), which meets our searching requirement of unknown environments, is referring to the work of Xu et al. [26].

**Figure 5:**
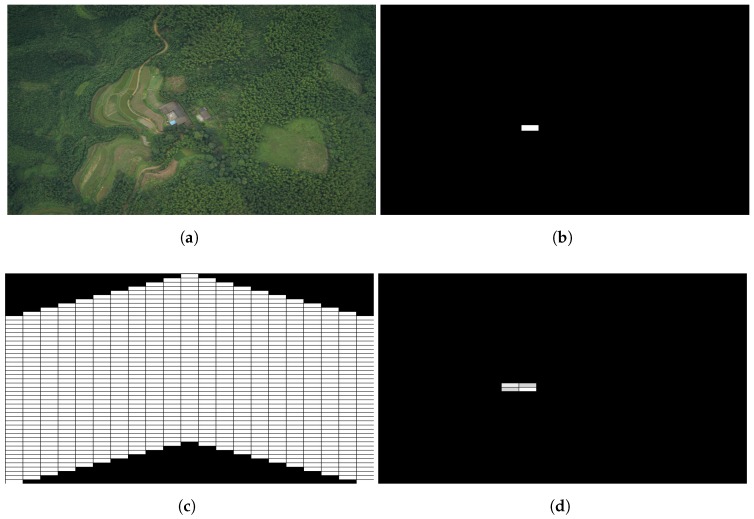
Simulated Distributed Salient Region Detection of Swarm Formations. (**a**) Original Scene; (**b**) Detection Result of Line Scan Case; (**c**) Coverage Area of V-Shape Scan Case; (**d**) Detection Result of V-Shape Scan Case.

By using the average intensity feature as the input of each member and applying (5)–(7), the salient region detection results are obtained, as shown in Figure 5.

Figure 5a shows an original scene. Start from the bottom row of the split scene with zero initial condition, the line formed swarm scan the whole scene line by line to the top. The state values of each block sampled by swarm members are calculated by applying (5), and then transmitted to the neighbors on left or right depends on the propagating causalities defined before. Based on the received state values, stored previous state values and the input (average intensity of the block) of each sample node, the output of each block is obtained by combining the outputs of both causalities. Figure 5b is the region saliency map obtained by putting the outputs to the corresponding location of a gray image and resize it to the same dimension as the original scene. We applied a discrimination threshold σ=20 to eliminate negligible values. We can see this method can detect the blue roof of a house from the original scene since it is more salient than the surroundings.

**Figure 6:**
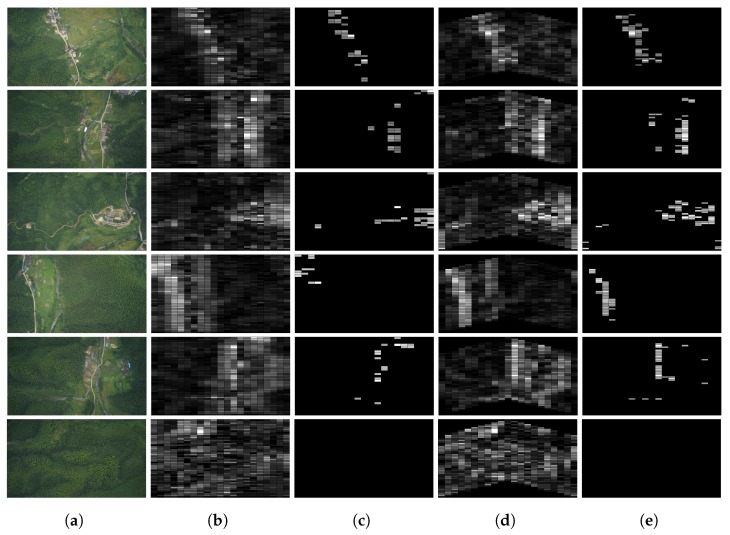
Formation Based Distributed Salient Region Detection. (**a**) Original Scene; (**b**) Line Case; (**c**) σ=20 (Line); (**d**) V-shape Case; (**e**) σ=20 (V-shape).

The simulation procedure of distributed processing for the v-shape case is similar to line formation case. The difference is that the horizontal states propagation route is bent which causes the coverage area change. The line case is able to cover the whole scene shown in Figure 4, while the coverage of the v-shape case is shown in Figure 5c, the white blocks are the covered area. Despite the swarm in this formation case have some blind area initially, it could be ignored in a long distance scanning. The salient region detection result of the same original scene is shown in Figure 5d, we can see it still holds the similar salient region location compares to the line case. More visual results are presented in Figure 6. Figure 6a shows the original scene of our simulation. Figure 6b,d demonstrate the processing results of line case and v-shape case respectively. Figure 6c,e are shown the thresholded results. The gray blocks indicate the salient regions in the scene, and the white one indicates the most salient region.

**Figure 7:**
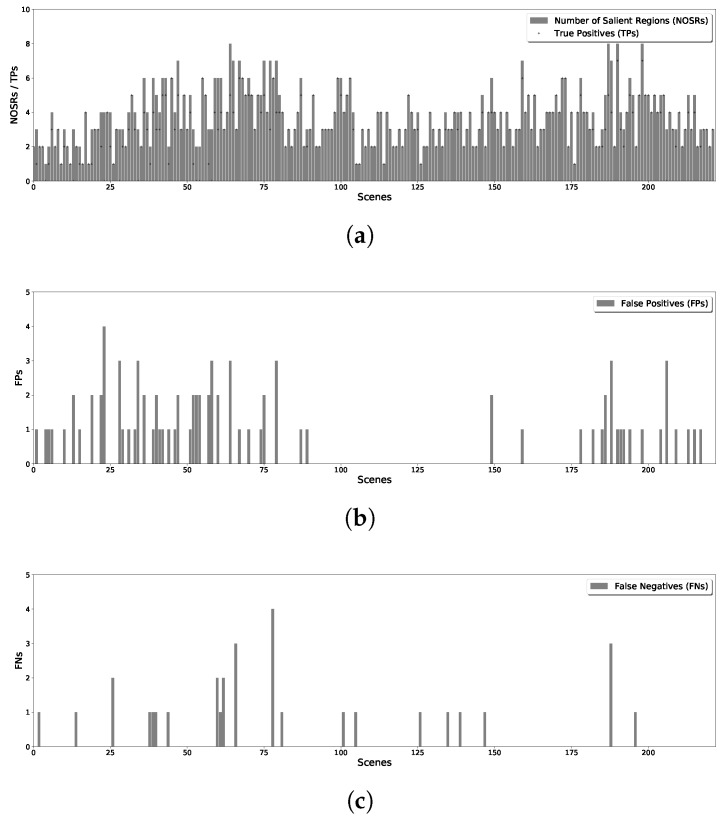
Statistical Results of Line Formation Case. (**a**) True Positives of Each Scene; (**b**) False Positives of Each Scene; (**c**) False Negatives of Each Scene.

**Figure 8:**
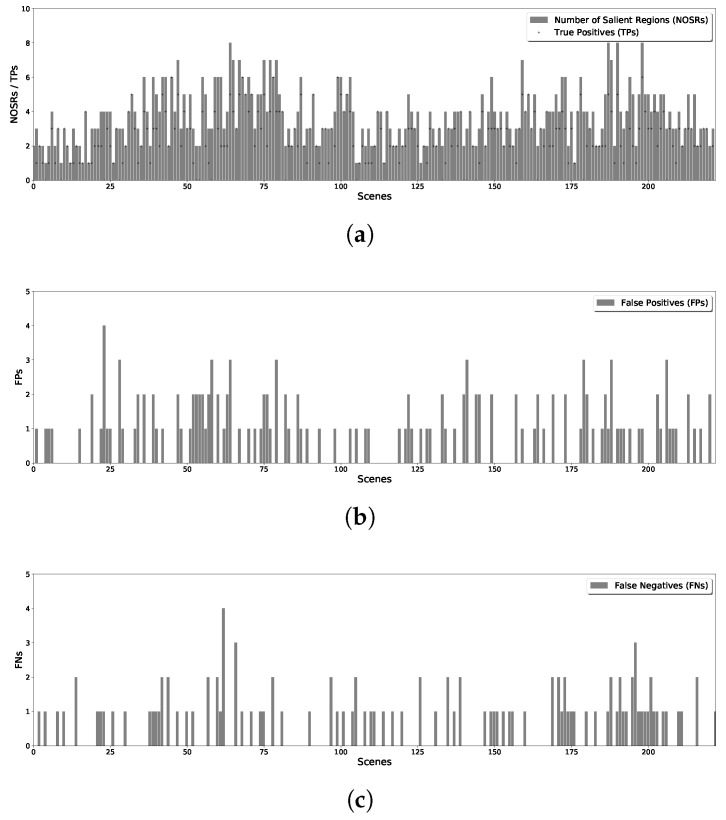
Statistical Results of V-shape Formation Case. (**a**) True Positives of Each Scene; (**b**) False Positives of Each Scene; (**c**) False Negatives of Each Scene.

By splitting all the images in the dataset, and running the proposed methods for line and v-shape case, we get the results of detected salient regions. With comparing with the results with manually labeled salient regions of original scenes, the statistical results of proposed methods are shown in Figure 7 for line case and Figure 8 for v-shape case respectively. Figure 7a and Figure 8a illustrate the number of salient regions (shown with the bars) and the detecting true positives (shown with the dots) in each scene. Figure 7b and Figure 8b give the number of false positives for line and v-shape case respectively. We can see the number of false positives in the v-shape case is higher than that in line case. Since the state propagation direction is bent, the reference blocks are not exactly left or right blocks. It makes the salient value is not the same as the line case, use the same threshold to segment the saliency map will get different results. Additionally, Figure 7c and Figure 8c show the number of false negatives of those two situations. This number of v-shape case is also more significant than line case because there are some blind areas of v-shape scanning. With long distance scanning, the blind areas can be ignored.

The average results of the above values are shown in Table 1. The recall rate and precision rate of line formation case is 95.77% and 88.96% respectively. Those rates are both higher than the v-shape case, which is 84.95% and 80.86% correspondingly. Since the salient region detection is typically used for rough inspection of an area, after this procedure, one may take other operations to the specific region for detail inspection. Under this consideration, those rates are acceptable for salient region detection tasks. This example evaluated proposed distributed framework is suitable for formation formed swarm tasks.

## 5. Discussion

It can be seen from the listed results above, the presented distributed processing method for collaborative salient region detection is proved to be effective with swarms in line or v-shape formations. Here are some more questions that have to be discussed.

### 5.1. Unbalanced V-Shape Formation

**Figure 9:**
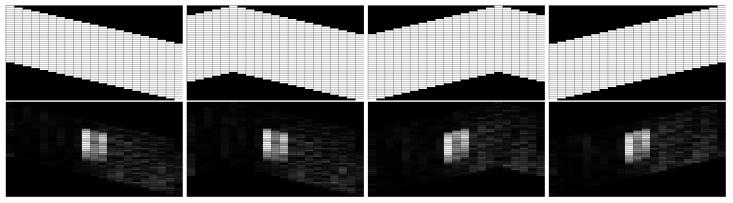
Distributed Salient Region Detection with Unbanlenced V-shape.

The final shape of the v-shape formation may vary, i.e., it may result in unbalanced formations, which will influence the coverage area of the swarm. As shown in Figure 9, we tested several unbalanced v-shape cases for our distributed salient region detection algorithm. The images in the first row are the coverage areas of different unbalanced formations. The images in the second row are the corresponding detection results. Although the coverage area has changed, the detected salient region is the same in those situations. That means the overall properties of the algorithm still hold.

### 5.2. Imperfect Formations

**Figure 10:**
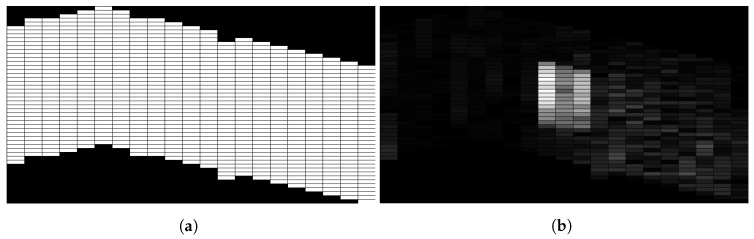
Results with Imperfect V-Shape Scan. (**a**) Imperfect V-shape Scan Coverage; (**b**) Processing Results.

Since the sensor zero drift or controller precision of the swarm member introduces position errors, it will result in imperfect formations. Figure 10 shows an example of processing with an inaccurate v-shape, we can see since the relative positions of members are not changing during flying, the salient region is still detected. Therefore, it can still hold the effectiveness under the condition of imperfect formations with position errors. The detected region is the same as in Figure 9.

### 5.3. Other Concerns

The proposed processing method is based on spatial related multidimensional system theory. The relative positions of members in the swarm are essential for processing. In the initial or transient phase of pattern formation, if the relative positions are totally disordered, the output of the method will be invalid unless the positions of every member in the swarm are recorded, and some recovery algorithms are applied. We can use other strategies to avoid this case, for example, forming the formation using a slower speed before reaching the interest area, or revisit the area after formation, etc. Furthermore, the proposed example of salient region detection we have shown is only able to detect static or slowly moving salient targets. Nevertheless, by applying other filters (e.g., velocity filter [27]) to the distributed processing framework, the introduced Roesser model based distributed processing paradigm has the capability to detect other events or dynamically changing signals.

## 6. Conclusions

This paper introduces a new distributed processing paradigm for formation formed robotic swarms in searching tasks. The in-swarm distributed processing is performed using a modified 2-D Roesser model with half-plan causality. The concept is illustrated using a line (or v-shape) scanning swarm that implements the 2-D algorithm sequentially, i.e., by executing the 2-D filtering algorithm line by line. The effectiveness of this framework is shown by an example of salient region detection task, making it a good candidate for collaborative sensing of robotic swarms.

## Figures and Tables

**Table 1:** Statistical Results.

	Line Case	V-Shape Case
No. of Scenes	222	
No. of Salient Regions	732	
True Positives (TPs)	701	621
False Positives (FPs)	87	147
False Negatives (FNs)	31	111
Recall Rate: TPs/(TPs + FNs)	95.77%	84.95%
Precision Rate: TPs/(TPs + FPs)	88.96%	80.86%
